# Epidural Fibrin Sealant Injection for the Management of Cerebrospinal Fluid Leak Following Dural Puncture in Children

**DOI:** 10.7759/cureus.6940

**Published:** 2020-02-10

**Authors:** Stephanie A Armstrong, Huy Tram N Nguyen, Susan L Rebsamen, Bermans Iskandar, James A Stadler

**Affiliations:** 1 Neurological Surgery, University of Wisconsin School of Medicine and Public Health, Madison, USA; 2 Radiology, University of Wisconsin School of Medicine and Public Health, Madison, USA

**Keywords:** csf leak, epidural blood patch, intracranial hypotension, fibrin

## Abstract

Cerebrospinal fluid (CSF) leak, intracranial hypotension, and postdural puncture headaches are common following dural punctures. Management usually consists of conservative treatments with medications (e.g. caffeine, nonsteroidal anti-inflammatory drugs, steroids, opioids), increased fluid intake, and bed rest. In more severe and persistent cases, epidural blood patches (EBPs) are indicated. When multiple EBPs fail, epidural injection of fibrin sealant has been successful in a few reported adult cases. The authors describe the first reported clinical experiences of epidural fibrin patch in children for repair of CSF leak and resolution of intracranial hypotension. This technique was used in three cases where serial EBPs failed to resolve symptoms related to intracranial hypotension following dural puncture. Following the procedure, each patient had resolution of their presenting clinical symptoms and radiographic abnormalities, and there were no noted complications. Epidural fibrin sealant injection is a reasonable option for relieving intracranial hypotension due to CSF leak following dural puncture in children.

## Introduction

Postdural puncture headache (PDPH) can occur in up to 37% of patients following lumbar puncture [1]. Symptoms typically occur within five days of the index procedure and are attributed to cerebrospinal fluid (CSF) leak at the dural puncture site leading to intracranial hypotension. This results in headaches that are classically orthostatic, worsens when sitting or standing, and improves with bed rest. Theories for the mechanism for PDPH are based on the Monro-Kellie doctrine, which states that within an intact skull, the cumulative volume of intracranial brain, blood, and CSF is constant. Therefore, when the volume of one component decreases, the remaining partial volumes provide compensation. In this instance, a decrease in intracranial CSF results in increased volume of low resistance structures such as the dural sinuses and cerebral veins [2-5]. Additionally, the differential pressure between the intracranial and spinal subarachnoid space results in caudal displacement of brain tissue, which is presumed to result in traction on pain-sensitive structures [2,4,6,7].

The treatment of PDPH is primarily conservative, including recumbent bed rest, medications (caffeine, nonsteroidal anti-inflammatory drugs, opioids, and steroids), and increased fluid intake. Mild headaches typically show improvement within a week of lumbar puncture. However, for persistent moderate symptoms or more debilitating symptoms, an autologous epidural blood patch (EBP) is the current standard therapy in relieving PDPH [2,4,6]. The documented success rate of EBPs is 70%-98% for first and second attempts, necessitating the need for alternative intervention in cases of treatment failure [4,6,8]. While epidural fibrin sealant injection has been reported in the adult population for select cases, the authors report on the first experience of using fibrin glue for CSF leak after dural puncture in three pediatric patients [9-11].

## Case presentation

Three consecutive pediatric patients were identified by retrospective analysis of an IRB-approved, prospectively maintained database. Each patient had clinical and radiographic findings consistent with CSF leak and intracranial hypotension following dural punctures, despite serial blood patches. Recorded information included patient demographics (age, sex), medical history, clinical presentation, and procedures performed. Postprocedural imaging and clinical outcomes were evaluated to determine treatment response.

Materials and Methods

**Figure 1:**
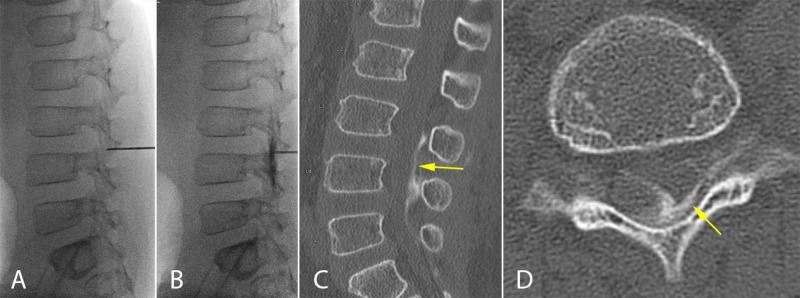
Epidural fibrin injection An example of fluoroscopically guided spinal needle placement (A) and confirmation with contrast (B) intraoperatively before injection of the fibrin sealant in Case 1. Postprocedural CT demonstrates appropriate distribution of the injection (C and D).

For each patient, epidural fibrin injection was performed jointly by a neurosurgeon and neuroradiologist. In all cases, the patients were in the left lateral decubitus, with hips and knees flexed. The spinal level was determined by clinical history and radiographic findings, targeting the best estimation of the site of the initial dural puncture. Fluoroscopy was used to identify appropriate needle positioning (Figure 1A-1D). Epidural positioning of the needle was confirmed fluoroscopically with injection of 1-2 cc of Omnipaque (GE Healthcare, Cork, Ireland) contrast. A fibrin sealant (TISSEEL, Baxter Healthcare Corporation, Deerfield, IL) was prepared and injected through the spinal needle. The stylet was reinserted, and the needle was removed. Bed rest was maintained immediately following the procedure, utilizing the same care as would follow an autologous EBP.

Three pediatric patients underwent epidural fibrin glue injections for CSF leak after dural puncture and subsequent failed serial blood patches. The patient histories were significant for medulloblastoma requiring CSF sampling, inadvertent dural puncture during administration of epidural anesthesia for an orthopedic procedure, and pseudotumor cerebri. The average patient age was 12.3 years, with two males and a female. The epidural fibrin injection successfully resolved CSF leak in these cases, with noted clinical and radiographic improvements in each patient. Debilitating symptoms of intracranial hypotension resolved within an average of 8.6 days postepidural fibrin patch.

Case 1

**Figure 2:**
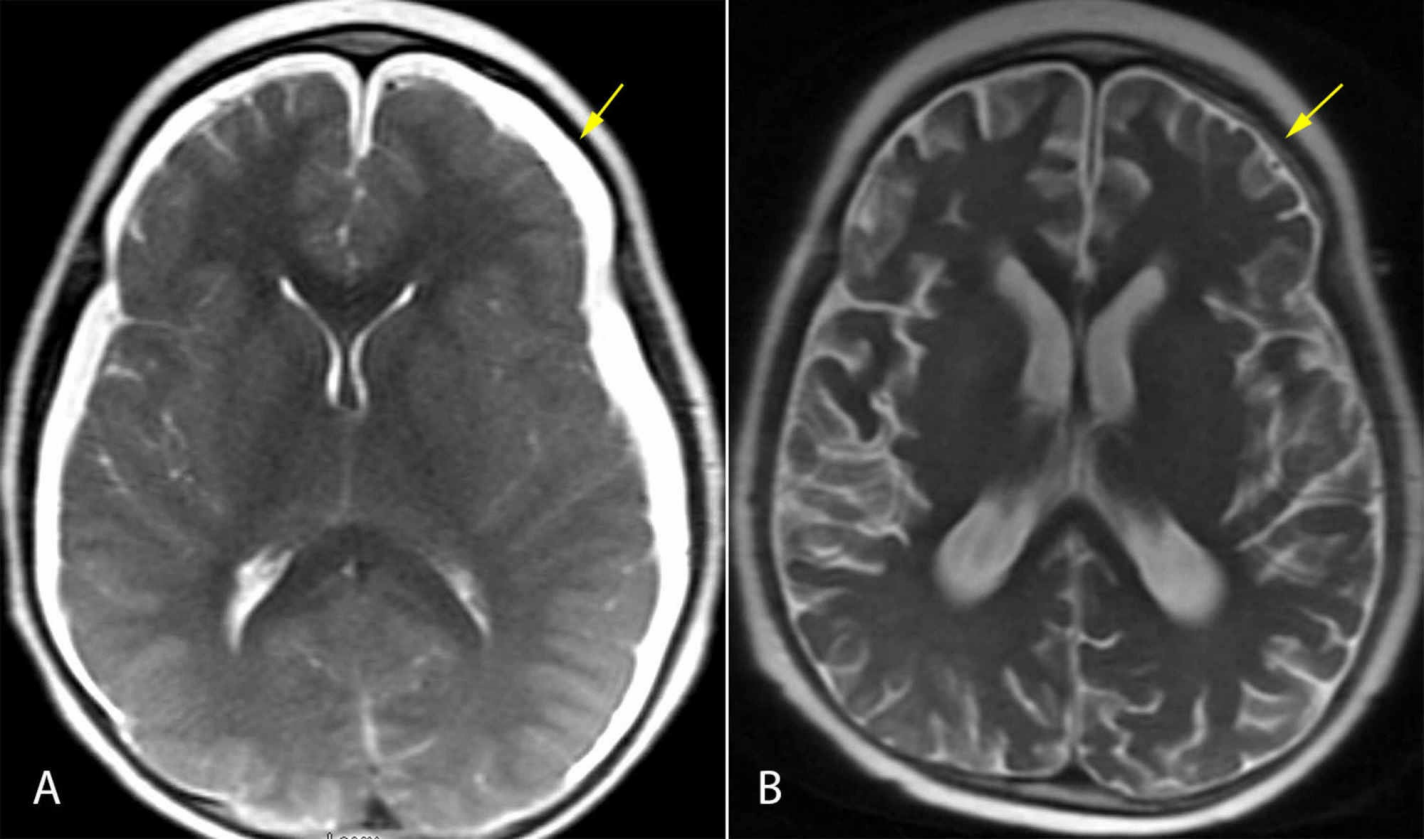
Case 1 Initial brain MR images demonstrating subdural fluid collections, ventricular compression, and caudal descent of the brainstem (A). These findings reversed postoperatively (B).

**Figure 3:**
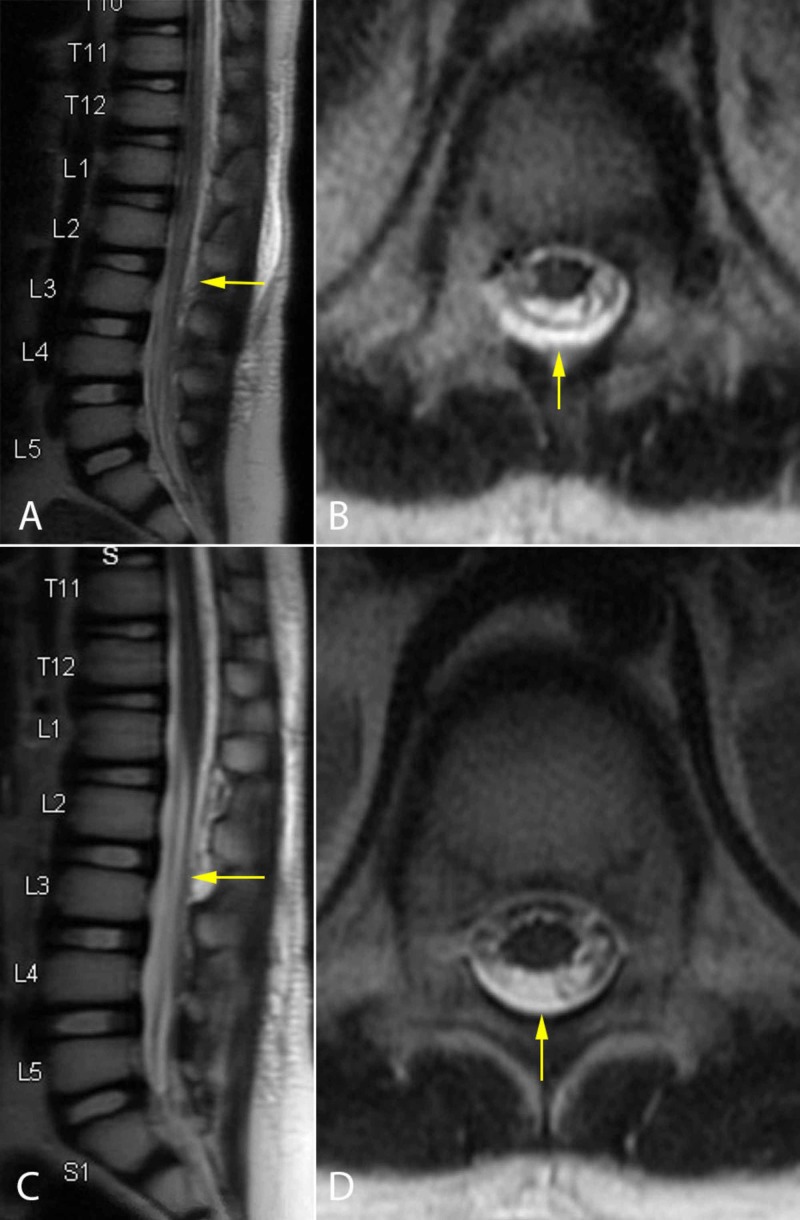
Case 1 Initial spine MR images showing the subcutaneous and epidural fluid collection (A and B). Postoperative spine MR images show resolution of the epidural fluid collection (C and D)

A nine-year-old male presented with positional headaches, nausea, vomiting, pruritis, and fever following a diagnostic L2/L3 lumbar puncture performed to evaluate for metastatic medulloblastoma. His history was significant for recurrent anaplastic medulloblastoma previously treated with radiochemotherapy, and secondary acute myeloid leukemia requiring stem cell transplant. Brain MRI demonstrated 8 mm thick subdural fluid collections bilaterally and caudal descent of the brainstem (Figure 2A). MRI of the spine revealed dorsal subcutaneous paraspinal fluid from T11 to L3 and epidural fluid collection from T1 to L4 (Figure 3A, 3B). Two separate EBPs were performed without improvement in his symptoms. Surgical intervention for CSF leak was contraindicated due to pancytopenia secondary to acute myeloid leukemia. The patient underwent a lumbar epidural injection of 10 cc fibrin sealant via an 18-gauge spinal needle guided by fluoroscopy. A small amount of contrast was co-administered to confirm distribution of the injection from L3-L5. Postprocedural brain MRI revealed resolution of brain slumping, subdural fluid, and deformation of the ventricular systems (Figure 2B). Postoperative spine MRI demonstrated interval resolution of the epidural fluid (Figure 3C, 3D). The positional spinal headaches improved within three days of the procedure, and he had complete resolution of symptoms two weeks later.

Case 2

A 14-year-old female presented with severe postural spinal headache and back pain following epidural catheter anesthesia placement for orthopedic hip surgery two months prior. Her history was significant for hypersensitivity to iodinated contrast. Three EBPs, a myelogram lumbar puncture, and conservative interventions including bed rest, caffeine, and increased fluids were attempted with no resolution of symptoms. MRI of the spine demonstrated a significant dorsal epidural fluid collection from C7 to L1 and a ventral epidural collection throughout the lumbar region. Additionally, there was irregularity of the right ligamentum flavum at L3/L4 and L4/L5. Fibrin glue injection was performed at the L2 epidural space. Her postprocedural course was notable for mild facial flushing and sensation of throat swelling despite preventative medical treatment for contrast allergy per institutional protocol; her allergic symptoms resolved with antihistamine administration. Her presenting severe headaches resolved within five days of the procedure. One month after the procedure, the patient had brief mild headaches when rising to stand, but she was otherwise asymptomatic. Within two months of the procedure, her symptoms were completely resolved.

Case 3

**Figure 4:**
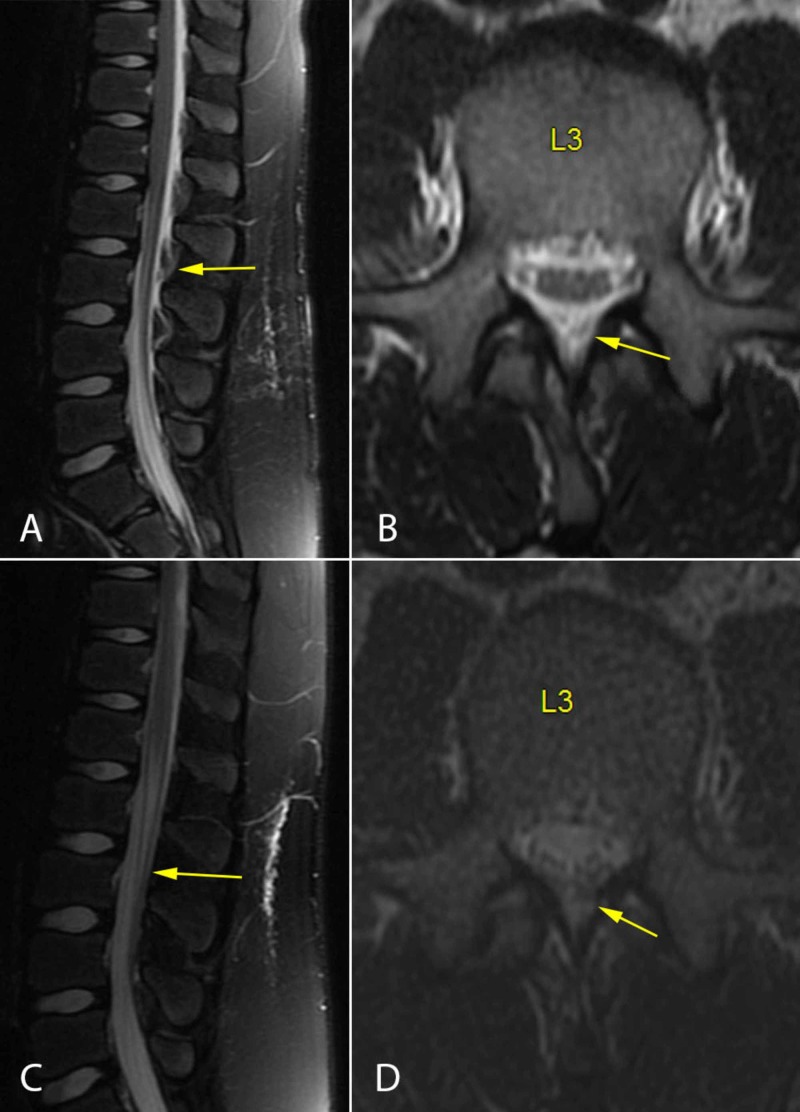
Case 3 Spine MR images showing the compressive epidural fluid collection before treatment (A and B), with resolution after treatment (C and D).

A 14-year-old male presented with intractable and positional retro-orbital headache, back pain, right lower extremity tingling, and decreased sensation to light touch following L4/L5 lumbar puncture for evaluation of pseudotumor cerebri. MRI of the lumbar spine demonstrated epidural CSF extending from the thoracic region to the lower lumbar spine; this resulted in mild deformation of the thecal sac (Figure 4A, 4B). Two separate EBPs were attempted with no improvement. A fluoroscopically guided epidural injection of 4 cc fibrin sealant was performed at L4/L5 using a 20-gauge needle. Postprocedural MRI showed resolution of the epidural fluid collection in the lumbar spine (Figure 4C, 4D). Clinically, he had resolution of the positional headaches within three days of the procedure and complete symptom resolution three weeks later.

## Discussion

Lumbar puncture is one of the most common invasive diagnostic procedures in children with corresponding PDPH occurring at a frequency of up to 4%-27% of patients [12-14]. During lumbar puncture, a lower number of puncture attempts, smaller needle sizes, a longitudinal orientation of the bevel, and at least four hours of bed rest following the procedure are correlated to lower frequency of the development of PDPH [13,15,16]. Using fluoroscopy during lumbar puncture decreases the occurrence of PDPH*.* Nonmodifiable risk factors include low body mass index, female gender, and previous history of PDPH [2].

Diagnosis of PDPH is, by definition, clinical. Symptoms suggestive of intracranial hypotension following lumbar puncture or other procedures that can result in CSF leak warrant appropriate management. Imaging is typically required only in patients with clinical ambiguous symptoms, more severe cases, or with failure of early treatments. MRI findings of intracranial hypotension include subdural fluid collections, caudal descent of the cranial contents, pachymeningeal enhancement, and venous engorgement. Spinal imaging often demonstrates epidural CSF collections; specific MRI protocols may increase sensitivity for the site of dural discontinuity [1].

When encountered, the management of PDPH starts with appropriate conservative measures. These include flat bed rest, hydration, medical pain management, and caffeine administration; however, these efforts have had variable rates of success [8,13]. EBP has typically been reserved for cases with failure of conservative management. The mechanism of dural sealing by EBP is still debated, with proposed theories of direct thecal tamponade and/or incitement of broader inflammatory processes that result in the closure of the dural defect [2]. Immediate effect may be related to compression of the dural sac decreasing its volume, while delayed effect is fibrosis or scar formation on dura. While EBP is the golden standard for cases of severe PDPH, alternative treatments should be considered when serial EBPs fail to resolve symptoms. While direct surgical repair remains an option if the defect can be confidently identified, this carries more significant risks and should only be considered in appropriate patients without less invasive options. Temporary CSF diversion requires either additional spinal dural puncture or ventricular puncture; permanent CSF diversion, such as with a ventriculoperitoneal or lumboperitoneal shunting, has both short- and long-term consequences [17,18].

Fibrin sealant is a common neurosurgical adjunct administered to enhance watertight dural closure with its high tensile strength and toleration of moist environments. The fibrin plug theory hypothesizes that adhesion created at the dural tear promotes healing by promoting the immediate stimulation of fibroblasts; direct tamponade likely provides additional effect [19]. Fibrin sealants have demonstrated success in treatment of CSF leaks following open surgery [10,19,20]. There is minimal risk of meningitis if the fibrin glue is introduced intrathecally; we mitigated this risk in our initial experience by using a small amount of contrast to confirm epidural positioning of the needle. Using fluoroscopy, a small volume of fibrin sealant can be effectively targeted to a specific dural puncture site, providing a less invasive management option for patients that have failed other efforts for control of a CSF leak and intracranial hypotension. The optimal volume needed for effective treatment is not known.

We describe here our initial experience using epidural injection of fibrin sealant for the treatment of PDPH in children. While this early case series is inherently small, the demonstrated success in patients that failed traditional conservative measures and multiple EBPs suggests this is a reasonable management option. We suggest that our findings be considered before initiating more invasive interventions, including direct surgical repair and CSF diversion.

## Conclusions

Intracranial hypotension after dural puncture remains a challenge for select pediatric patients. In cases with failure of conservative management and EBPs, epidural fibrin sealant injection is a reasonable option. Our early experience is presented, with successful management of three pediatric patients with difficult and persistent PDPH symptoms and intracranial hypotension. 
